# Staff Enablement of the Tovertafel for Enrichment in Residential Aged Care: Field Study

**DOI:** 10.2196/67919

**Published:** 2025-08-12

**Authors:** Ryan M Kelly, Asmita Manchha, Jenny Waycott, Rajna Ogrin, Judy A Lowthian

**Affiliations:** 1 School of Computing Technologies RMIT University Melbourne Australia; 2 School of Computing and Information Systems The University of Melbourne Melbourne Australia; 3 Silverchain Melbourne Australia; 4 Bolton Clarke Melbourne Australia; 5 School of Public Health and Preventive Medicine Monash University Melbourne Australia; 6 Faculty of Health and Behavioural Sciences The University of Queensland St Lucia Australia

**Keywords:** aged care, care staff, creative care, older adults, play, residential aged care, social enrichment, technology, Tovertafel

## Abstract

**Background:**

Enrichment activities are essential for enhancing the psychosocial well-being of older adults living in residential aged care homes. There has been increasing interest in using digital technology for enrichment, but the implementation of technology requires careful support and enablement from staff to ensure that residents experience the intended benefits.

**Objective:**

This study aimed to understand how care staff facilitate aged care residents’ use of the Tovertafel (“magic table” in Dutch), a technology that projects images onto a tabletop to enable groups of people to play games. The study further aimed to understand the benefits arising from the Tovertafel when facilitated by staff.

**Methods:**

We conducted a field study in 1 residential aged care home in Queensland, Australia. The methods included semistructured interviews with the staff and residents about their experiences with the Tovertafel, observations of 4 sessions in which the residents and staff played Tovertafel games, and a diary completed by the staff after Tovertafel sessions. Data were analyzed through reflexive thematic analysis.

**Results:**

We developed 3 themes through our analysis. Theme 1 highlights the need for the staff to overcome physical and personal barriers before Tovertafel sessions could take place. These included a lack of a dedicated space for playing Tovertafel games and the residents’ reluctance to attend Tovertafel sessions. Theme 2 highlights how the staff used creative strategies to make Tovertafel sessions successful. These included helping the residents learn how to interact with the games; adapting the activity to suit the capabilities of the residents; sustaining engagement by choosing appropriate games; and using prompts, questions, and storytelling to make the games more engaging. Theme 3 describes the benefits and outcomes that arose from staff-supported enablement of the Tovertafel, including participation in an enjoyable physical activity, socialization, and reminiscence.

**Conclusions:**

This study suggests that the Tovertafel provides opportunities for aged care staff to engage in creative play and personalization catering to residents with different capabilities. However, the benefits arising from the Tovertafel are unlikely to be achieved without substantial facilitation from the staff, who play a key role in enabling the participation of the residents. Sustaining the engagement of the residents is important during Tovertafel activities and can lead to beneficial outcomes.

## Introduction

### Background

In 2023, a total of 185,000 Australians aged ≥65 years lived in residential aged care homes [1]. Older individuals usually move to residential care when they require high levels of support to enable appropriate clinical care alongside holistic well-being [2,3]. Holistic well-being refers to addressing residents’ physical, psychological, and social needs [4]. To promote physical well-being, staff members from lifestyle teams support residents with activities of daily living, such as bathing, dressing, and eating, and enable them to participate in physical activities through exercise programs.

To promote psychological and social well-being, staff members provide an array of enrichment activities, such as art, gardening, music, games, and excursions. These activities are important for encouraging social interaction and preventing boredom [5], enhancing residents’ quality of life [6], and promoting a sense of continuity, particularly when residents engage in activities that interest them [7]. However, activities in residential care are often group based and may not cater to the needs of all residents [6], particularly when there is diversity among residents regarding individual interests [7], cultural background [8], or functional and cognitive capabilities [9,10].

Recent years have seen an increasing uptake of digital technologies for enrichment in residential care homes [11]. Technology-based activities can both expand the range of available options and enable new experiences of joy and social engagement [12]. For example, immersive virtual reality (VR) offers the possibility of virtually “traveling” to distant locations, revisiting places from the past, and remotely attending concerts [13,14]. When used appropriately and in a way that demonstrates person-centered care, technology-based activities can address the individual needs and goals of residents, including those with cognitive impairment [11].

However, research has identified that appropriately trained staff are often required to enable the effective use of technology in aged care [15]. Residential care staff are known to be dealing with high workloads [16], and there is a risk that facilitating technology may add to this load. While technology facilitation can be enriching when it involves meaningful care work [17], significant work in setting up and manipulating the technology can be an unwanted burden that limits the use of the technology [18,19]. Therefore, evaluating the demands placed on care staff, along with the potential benefits of using technology with residents, is an important consideration when justifying the use of technology as part of a care home’s lifestyle and social calendar.

### The Tovertafel

This study focuses on staff involvement in enabling the Tovertafel (“magic table” in Dutch), a commercial system that enables groups of people to play games by tapping on moving images projected onto a tabletop [20]. The games vary in difficulty, from simple activities such as sweeping leaves or popping bubbles to more advanced puzzles and team-based games that require cognitive and social engagement. While the Tovertafel is aimed at providing stimulation for care home residents living with dementia, it can be used by residents with various levels of cognitive impairment. Table 1 provides examples of Tovertafel games.

**Table 1:** Examples of Tovertafel games.

Game name	Description
Leaves	Players use arm movements to sweep piles of leaves from the tabletop.
Football	Players try to score goals at either end of the table by tapping a soccer ball.
Music Box	Players tap on musical notes rotating around a music box in the center of the table. The notes play piano sounds when tapped. At the end of the game, the music box reveals a spinning ballerina and plays a song.
Rhymes	Players tap on feathers to reveal the words of a nursery rhyme, which can then be read aloud.
Sayings	Players tap on moving bubbles to reveal a phrase or saying, for example, “Never look a gift horse in the mouth.”
Silverware	Players wave their arms over images of cutlery to simulate polishing a dinner set. Once all pieces are polished, plates and food appear on the tabletop, simulating the table being laid at dinner time.
The Veggie Patch	The game simulates gardening by requiring players to plant and water vegetables. Seed packets move over the table, causing vegetables to be planted when tapped. There are also moving clouds and sunbeams, which cause the plants to grow when tapped.

A small number of studies have examined the use of the Tovertafel in residential aged care. These studies have primarily focused on identifying benefits and drawbacks of the technology. Good et al [21] interviewed care staff about the perceived benefits of the Tovertafel for residents living with moderate to advanced dementia. Staff felt that using the Tovertafel had several benefits, including improved communication between staff and residents and short-term improvements in residents’ mood. However, the Tovertafel was rarely used by residents without initiation from carers or loved ones, and residents with dementia could only use the Tovertafel for a short time because of impaired concentration. A study by Talman and Gustafsson [22] identified benefits of the Tovertafel for people living with intellectual disabilities. However, these findings may not be applicable to aged care. The Tovertafel’s official website lists various studies that have been conducted to validate the system, but most are unpublished, originate from master’s theses, or require readers to contact the company to obtain further information [23]. Other than the studies mentioned earlier [21,22], the only publicly available study evaluating the Tovertafel is in Dutch [24].

While the impact of the Tovertafel may be positive, there has been limited examination of the care strategies required to facilitate the participation of residents in the activity. There is also limited understanding of how staff work to overcome issues that may impact the engagement of aged care residents with the Tovertafel. Research is needed to better understand how staff facilitate the Tovertafel activity and to inform decisions about using this technology, given the limited resources available in many residential aged care homes.

### Objectives

This study aimed to address the following research questions:

1. How do care staff enable residents’ use of the Tovertafel in residential aged care homes?
2. What benefits arise from staff-supported enablement of the Tovertafel?

## Methods

### Ethical Considerations

All procedures in this study received approval from the University of Melbourne’s Human Research Ethics Committee (ID 12900) and the Bolton Clarke Human Research Ethics Committee (approval 230001). Our study involved participants who were either staff or residents from one aged care home in Australia. The staff participants signed a participant information sheet and consent form (PICF) and received an Aus $20 (US $13.37) gift voucher as a token of appreciation.

The care home residents who participated in the interviews were provided with a PICF that included pictures of Tovertafel games, along with a textual description of the technology. Some of these residents were shown a YouTube video of the Tovertafel if they were unable to understand its functionality from the PICF alone. The residents who agreed to be interviewed provided informed consent by signing the PICF and received baked goods as a token of gratitude. Some residents living in the memory support unit of the care home did not have the capacity to consent, so interviews were not sought about their experiences with the Tovertafel. However, we observed the sessions in which these residents participated and collected observational notes about their use of the Tovertafel, without capturing personal or identifying information, in line with the ethics approvals for the project.

All data from the study was storied securely in password-protected filing cabinets or secure cloud-based storage to protect the confidentiality of the data. All transcripts were anonymized, and names were replaced with pseudonyms to protect participants’ privacy.

### Study Design

We conducted a field study to investigate how the Tovertafel was used in 1 Australian residential aged care home. The study involved semistructured interviews, observations, and a participant diary. We chose these methods to gain in-depth insight into staff members’ experiences of facilitating the Tovertafel and to understand how this facilitation elicits benefits for residents. All procedures were developed by the research team, drawing on our previous experience with the chosen methods [25,26] and our experience of conducting fieldwork in aged care [8,27,28].

First, the staff who were familiar with using the Tovertafel were interviewed about their experiences of using the system, the strategies that they had used to make the activity successful, and the perceived benefits. We also conducted interviews with the residents who had used the Tovertafel, provided they had the capacity to give informed consent and provide feedback. These interviews sought to understand what the residents liked about the activity and what they found challenging. The interview schedule for the residents contained simple questions to avoid overburdening them.

Second, we observed 4 activity sessions, each lasting 45 minutes, in which small groups of staff and residents played a variety of Tovertafel games. These sessions were scheduled by the lifestyle team of the care home as part of the usual activity schedule. The observations enabled us to understand how the Tovertafel was used in practice, what the staff did to make each session successful, and how the residents responded to the activity.

Third, the staff leading the Tovertafel activity were asked to complete a diary after each session. The diaries invited further reflection on the use of the Tovertafel, including the views of the staff about the impact of the Tovertafel on the mood of residents, what worked well, and what the staff found challenging about the session (refer to Multimedia Appendix 1 for the diary design). The staff were asked to complete diary entries after each Tovertafel session to capture their experiences as close to the time of delivery as possible.

Table 2 lists the observed Tovertafel sessions, the number of people involved, and the timing of diary entries completed by the staff members.

**Table 2:** List of the Tovertafel sessions examined.

ID	Date and time	Location	Number of people involved	Observed by researchers	Diary entry completed by staff
1	June 6, 2023, Tuesday, 9:15 AM-10 AM	The memory support unit	2 residents, 2 lifestyle staff, and 1 nursing staff	✓	
2	June 7, 2023, Wednesday, 9:15 AM-10 AM	The memory support unit	4 residents and 2 lifestyle staff	✓	✓
3	June 12, 2023, Monday, 9:15 AM-10 AM	The memory support unit	7 residents, 2 lifestyle staff, and 1 nursing staff		✓
4	June 21, 2023, Wednesday, 1:30 PM-2:15 PM	The nursing home	4 residents and 1 lifestyle staff		✓
5	June 27, 2023, Tuesday, 9:15 AM-10 AM	The nursing home	3 residents and 2 lifestyle staff	✓	✓
6	June 28, 2023, Wednesday, 9:15 AM-10 AM	The nursing home	6 residents and 2 lifestyle staff	✓	✓

### Study Site

Our study took place at 1 residential aged care home of a not-for-profit aged care provider in Queensland, Australia, in 2023. The home provides care for approximately 80 residents divided across 3 residential wings: a memory support unit for residents with severe cognitive impairment, a nursing home area for other residents with high care needs, and a hostel area for residents with lower care needs. The home had 112 staff at the time of our study, including, for example, administrative staff, nurses, managers, and housekeeping staff. The home had 2 staff members who oversaw lifestyle activities: 1 diversional therapist and 1 lifestyle activities coordinator. These staff members were supported to run activity sessions by nursing and personal care staff, depending on availability and the number of people required.

The residents at the home predominantly follow a daily routine with fixed times for meals and personal care. Enrichment activities are scheduled around this routine. The lifestyle activities coordinator prepares a monthly activities calendar, which is distributed to residents on paper. Each day has an activity in the morning and another in the afternoon. Activities last between 45 and 60 minutes, and the residents are free to attend as they please. All activities are announced by staff over a public address system transmitted across the 3 residential wings.

The care home has a large room for providing lifestyle activities. At the time of our study, this room was undergoing renovation. Because of this, the equipment for the Tovertafel had been installed in the dining areas of the memory support unit and the nursing home.

During this study, the Tovertafel activity was scheduled in the activities calendar, allowing the residents from the memory support unit and the nursing home to participate (Table 2). The residents from the hostel area were also welcome to participate. The staff had been using the Tovertafel for 2 years, having received the device as a gift from a benefactor in 2021, but it had not been included in the activities schedule for >6 months before this research. The staff had not received formal training on how to use the Tovertafel. Instead, they learned on the job. After the Tovertafel was first installed in the home, the diversional therapist downloaded a PDF of the system’s user manual to understand how the device should be operated.

### Participant Recruitment

The staff members were invited to participate in the study if they were familiar with the Tovertafel or if they were scheduled to help facilitate the sessions during the study period. All 6 staff members who met either of these criteria agreed to participate. Volunteers and family members were also eligible to participate in the study, but none were present when data were collected.

### Procedure

All procedures were carried out by 2 researchers (RMK and AM). Two fieldwork visits, each lasting 4 days, were conducted in June 2023. Both researchers spent several days on-site before and after the Tovertafel sessions, providing opportunities to learn about the social routines of the care home [29]. The researchers took photographs of the setting, without capturing images of the residents or the staff, and tested the Tovertafel games to understand what they involved. The dates and times of the observed sessions are provided in Table 2. The researchers created private, written fieldnotes after each day of the study to record their experiences and personal reflections about the care home.

During the first fieldwork visit, the researchers interviewed the staff members about their experiences with the Tovertafel and observed 2 Tovertafel sessions in the memory support unit. The researchers conducted nonintrusive observations of each session, observing the staffs’ and the residents’ interactions around the Tovertafel and with each other, from 2 to 3 meters away. The researchers took notes about which games were played in each session, what the staff did to facilitate the activity, and how the residents responded to the Tovertafel, including emotional responses and social interactions. All observations of emotional responses were noted in a free-text form, rather than using a predefined behavioral grid. The staff member in charge of each session was requested to fill out the research diary after they had facilitated the activity. All diary entries (5 in total) were completed by the coordinator of the lifestyle activities in the care home, who attended all the Tovertafel sessions examined during the study.

The first fieldwork visit was followed by a 2-week gap during which the researchers were off-site. Two Tovertafel sessions were scheduled by the staff during this time (Table 2). These sessions were not observed by the researchers, but the lifestyle activities coordinator completed the diary after each one.

During the second fieldwork visit, the researchers conducted observations of 2 Tovertafel sessions in the nursing home and invited the residents to provide feedback through interviews after each session. Follow-up interviews were conducted with the staff who had facilitated the sessions.

### Analysis

Data consisted of interview transcripts, observational and fieldwork notes, photographs, and diary entries. Interviews were transcribed using a professional transcription service. Data were analyzed using the 6 phases of the approach to reflexive thematic analysis by Braun and Clarke [30]. Reflexive thematic analysis is interpretivist and acknowledges that the subjectivity of the researcher can be a valued analytical resource, while also recognizing that the analysis is influenced by the positionality of the researcher [31]. This analysis was led by RMK, whose position is that technology has the potential to enrich the lives of people living in residential care but risks becoming an unnecessary burden, particularly if the technology is not well designed for this setting [17,26]. This position informed the analysis by highlighting both the burdens involved in enabling the Tovertafel and the enriching aspects of the work, particularly when it provided the staff with opportunities to enhance the activity for the residents.

Following the 6-phase approach by Braun and Clarke [30], RMK first read through the data before inductively creating semantic and latent codes at the sentence level [31]. RMK collated the codes into potential themes that captured interpretations of the problems the staff had faced, the ways that they facilitated sessions, and the perceived benefits of the Tovertafel. The analysis was discussed several times with AM, who iterated the themes based on her involvement in the data collection. RMK then drafted the themes into an initial version of the paper, which was discussed and reviewed by all authors [32]. This team input supported a critical and balanced perspective on the Tovertafel activity. After receiving feedback from peer reviewers, the themes were revised and reorganized to strengthen the interpretations.

In the following section, we present 3 themes. These are organized chronologically, as the themes we developed align with a before-during-after framing of the activity and reflect the temporal ordering of actions conducted by the staff when facilitating the Tovertafel sessions. The first theme highlights the need for the staff to overcome physical and individual barriers *before* initiating the Tovertafel activity. The second theme reveals the importance of creative care from the staff *during* Tovertafel sessions. The third theme details the benefits and outcomes *arising from* staff-supported facilitation of the Tovertafel. Verbatim quotes are used to illustrate findings, with pseudonyms and roles at the care home used to identify participants. Descriptions of the residents’ emotions or reactions to the activity are based on observations by the researchers or entries made in the staff diaries.

## Results

### Participant Characteristics

All 6 staff members interviewed were women. The interviews lasted between 15 and 38 (mean 30, SD 8.7) minutes. Table 3 lists participants’ pseudonyms, roles, and time at the workplace for the staff participants. Each Tovertafel session was led by Corina, the lifestyle activities coordinator, along with at least 1 other staff member.

**Table 3:** List of interviewees.

Pseudonym	Gender	Role	Time at the workplace	Time in current role	Interviewed during the first fieldwork visit	Interviewed during the second fieldwork visit
Anne	Woman	Care home manager	20 y	4 y	✓	
Brenda	Woman	Diversional therapist	11 y	4 y	✓	✓
Corina	Woman	Lifestyle activities coordinator	4 mo	4 mo	✓	✓
Diane	Woman	Special care unit nurse	16 y	16 y	✓	
Ellen	Woman	Personal care worker	11 y	11 y	✓	
Fiona	Woman	Enrolled nurse	8 y	3 y	✓	
George	Man	Nursing home resident	N/A^a^	N/A		✓
Herbert	Man	Nursing home resident	N/A	N/A		✓
Ingrid, Julie, and Karen	Women	Hostel residents (interviewed together)	N/A	N/A		✓

^a^Not applicable.

In addition, 5 residents (2 men and 3 women) were interviewed after the Tovertafel sessions. These interviews lasted between 10 and 19 (mean 14.3, SD 4.5) minutes. Other residents were approached for the interview but either declined to provide feedback, were unable to participate because of severe communication impairments, or were occupied with personal care activities.

### Theme 1: Initiating Tovertafel Sessions Required Attending to Physical and Personal Barriers

#### Summary

Our analysis showed that the staff needed to overcome multiple barriers before running the Tovertafel activity. These included the lack of a dedicated space for play and the need to address residents’ uncertainty about joining the activity. These barriers reflect the physical and personal challenges associated with using group-based digital technology, such as the Tovertafel, within residential care homes. We provide further explanations of physical space and perceptions of technology as subthemes in the following sections.

#### Physical Space: The Care Home Lacked a Dedicated Space for Play

At the time of our study, the care home lacked a dedicated activities space because of an ongoing renovation in the activities room. All group activities had to take place in other communal areas.

Equipment for the Tovertafel system, including an electrical connection and a ceiling bracket, had been installed in the dining areas of the memory support unit and the nursing home. These areas were typically set up with tables and chairs in preparation for mealtimes. To make each space suitable for the Tovertafel activity, the staff had to reconfigure the furniture to create a larger table suitable for the residents to sit around.

The staff did not feel that this practical work was especially burdensome, although it did require additional effort. As part of the setup, the staff also placed a white cloth over the table for use as a playing surface, after noting that the games were hard for some residents to see without using the tablecloth. This was because the wooden tabletops had not been designed for use with the Tovertafel and tended to absorb the projected images, making a lighter surface necessary. In addition, the dining areas had large windows that let in considerable sunlight, making it harder for residents to see the projections if the tablecloth was not used:

The setup doesn’t take that long...but one thing I found was that the graphics were a bit too light. So maybe it needs to be a certain colour that it projects onto. Because I noticed that with a few of the games, [the residents] did struggle to see what it was.

Diane, care nurse

The residents noted that they were hesitant about attending Tovertafel sessions because of the lack of a dedicated space for play. As noted earlier, the activities room at the care home was undergoing renovation. The residents from the hostel area where the residents had lower care needs—were invited to join the activities that took place in the nursing home and memory care unit. However, they expressed reluctance to enter these areas and felt that that it would be better if the Tovertafel was in a shared activities room:

I was wondering if it’s possible to have that in the activity room. More people might go then...a lot of people down here don’t like going into the nursing home.

Julie, hostel resident

In addition to needing to overcome physical space barriers to initiate the Tovertafel sessions, the staff also had to ensure that they were able to reset the space at the end of each session. We observed that there was a risk of conflict if the chairs and tables were not rearranged in the same order as before. This was because specific residents could not be seated together, and the staff needed to place the tables carefully to minimize extra work for the care and culinary staff during mealtimes. This situation also meant that the Tovertafel could not be left available for the residents to use at their convenience. As highlighted in previous work [21], the staff thought that the system might be used more often if residents could use it with visiting family members, without the need for staff assistance.

#### Perceptions About Technology: Residents Were Often Reluctant to Join Tovertafel Sessions

A second barrier that the staff had to overcome was the reluctance of the residents to join the sessions. The staff found it challenging to encourage the residents to attend the Tovertafel sessions because many were not interested in technology-based activities:

I find they’re not really interested in technology as a lot of the residents have farming backgrounds. They were used to milking the cows and doing all the farm work.

Ellen, personal care worker

People tend to go “under cover” when you mention the word technology.

Corina, lifestyle activities coordinator

In addition, the residents were often uncertain about what the Tovertafel involved if they had not seen it before. Most of the residents we encountered spoke English as their first language, but the word “Tovertafel” lacks obvious meaning for those who do not speak Dutch. The staff found that describing the Tovertafel as “the table game” or “something you play with your hands” was more effective, though still challenging without demonstrating the activity in person.

Because of this, the staff sought to increase attendance by going to the rooms of the residents before sessions took place to explain what the activity would involve and to assure the residents that it would be enjoyable. The staff also approached the residents who were in the nearby common areas when the activity began and asked them if they would like to give the activity a try. The staff felt this was different from other frequently facilitated activities (eg, choir and concerts), which the residents did not need encouragement to attend.

The issue of attendance was important because the staff felt that the Tovertafel worked best with groups of 4 to 6 players. Having 4 players was seen as too few to make the games enjoyable. This meant that achieving enough players was important for the activity to be successful. The staff told us that the Tovertafel had not been used for some time before the study because attendance had been poor:

We have used it before, but we reached a stage where we weren't getting a lot of interest. So, I’ve taken it off the activity calendar for a time. We just weren’t having the numbers turn up to the activity.

Brenda, diversional therapist

The staff were also conscious that running the activity too often with the same people risked creating disinterest. This view was echoed by the residents:

I think if we played it every day, it might get boring. Once a week, that’s no problem.

George, nursing home resident

To initiate the Tovertafel activity, the staff needed to be aware of which residents were available to join, how many were willing to participate, and had to encourage those who were available to attend. Addressing these social dynamics was challenging and time consuming but was essential to ensure that sessions had sufficient participants to make the games worth playing.

### Theme 2: The Staff Used Creative Care Strategies to Guide the Tovertafel Sessions

#### Summary

This theme highlights how the staff creatively enabled the use of the Tovertafel *during* the activity; that is, once the staff had created the conditions for a Tovertafel session by arranging the play area and encouraging the residents to attend, additional work was needed to make the sessions successful. We observed that the staff used creative approaches to help residents learn the activity, enable the continued participation of the residents, and sustain their engagement. Each of these activities required differing forms of creativity, which are described in the subsequent sections.

#### Selecting Gentle Games to Enable Initial Learning

At the start of each session, we observed that the residents were typically unsure what the Tovertafel was and what it involved. In 1 diary entry, Corina, the activities coordinator, similarly noted that the residents were hesitant at the beginning of a Tovertafel session but became more confident once they understood what to do:

The mood was a bit daunting and [residents were] unsure what was going on but once we got started the mood changed and [they] became interested.

Corina, lifestyle activities coordinator, diary excerpt, session 2

This highlighted the need for the staff to help the residents learn how to use the Tovertafel. The staff had identified a creative way to achieve this learning. At the start of each session, they chose to leave a simple game running on the tabletop while helping the residents join the activity. The staff typically chose the game Leaves for this situation. This game involves sweeping leaves from the tabletop using arm movements. We observed that playing with the leaves helped the residents to understand what would happen if they touched the images. Changes in the leaves were both noticeable and easy to trigger, helping the residents understand how the Tovertafel reacts to their movements.

In this way, starting each session with a gentle game provided opportunities for social learning, whereby the residents learned about the activity through watching others. In session 2, we observed a resident who repeatedly exclaimed that she could not join in because she “didn’t know what it was all about.” She eventually began playing after watching the interactions of other residents and after receiving encouragement from staff.

These instances highlight that creative work was needed to make the residents feel comfortable at the outset of each session. They also reveal that the staff were not simply supervising the activity. Rather, they were central to making the residents feel at ease and actively encouraged the participation of the residents during the sessions.

#### Developing Creative Solutions to Ensure Safe Participation

Creativity was evident in the way that the staff developed solutions to other problems that hindered the participation of the residents. For example, some residents found it challenging to trigger a response from the Tovertafel because of mobility impairment. Many could not stand up without assistance and therefore could not reach across the table, while others had difficulty moving their hands or arms.

Recognizing this, the staff had cut up a “pool noodle” (ie, a cylindrical flotation device made of foam) into pieces, which the residents then held to extend their reach and trigger the Tovertafel more easily:

Some [residents] have problems with their arms, or they’ve hurt their elbow in some way. So having the pool noodle, they don't have to reach. And a lot of residents tire very quickly when they're using the arms.

Brenda, diversional therapist

The staff were conscious that some residents might experience motion sickness when using the Tovertafel. In session 2, the staff paid close attention to a resident who was known to experience vertigo in response to moving images. The staff described the importance of attending to visual cues, such as body language and facial expression, while running the game to ensure that the residents remained safe. In this way, the staff exhibited creative care by monitoring for signs of distress during the games and changing to a simpler game if required:

You need to go by the visual cues and that’s probably the advantage of Tovertafel. It’s a visual reaction. And it can depend day to day on that resident, with how they’re going to interact in an activity or with the Tovertafel.

Anne, care home manager

#### Sustaining Engagement by Choosing Appropriate Games

The importance of choosing games was mentioned repeatedly by the staff during the interviews. We observed that the staff always took responsibility for selecting games, using a remote control to pick games from the Tovertafel’s menu system.

The staff explained that choosing appropriate games was essential to sustain the engagement of the residents. One reason was that the staff felt that there needed to be a good match between the games on offer and the physical or cognitive ability of the residents in attendance:

[It] depends on who you’ve got present in the session, because they’ve all got varying attention spans.

Anne, care home manager

In addition, the games needed to be relevant to the interests of the residents; otherwise, the sessions risked becoming boring. The staff explained how this risk could be mitigated by choosing games with features that appealed to the residents. Some Tovertafel games replicate tasks such as gardening and cooking. These games were perceived as engaging because they are modeled on familiar leisure activities. For instance, the staff often selected the Veggie Patch game because many residents were interested in gardening:

The gardening game is one where many of the ladies enjoy gardening, so we will do that and ask what they used to do in their garden. There’s one person who got very engaged by that. She loves gardening and that’s one way to try and get her to engage in the game because gardening was her hobby.

Brenda, diversional therapist

Sometimes the staff chose games that were known to be exciting and avoided games that they thought were boring. This was established through trial and error, as well as through verbal and nonverbal feedback from the residents. The staff also switched between games based on the current state of the residents. For example, if residents appeared to be losing interest in a gentle game, staff would switch to one involving physical activity to help engage them:

I find if they play it for too long, they get a bit tired of it and they get a little bit bored. So that’s why you’ve got to change to a different game, just to change the mood. It seems like they like something a bit quicker as well, more so than something that’s a little bit slow and repeated.

Corina, lifestyle activities coordinator

Finally, the staff had identified that staying with the same game for too long risked creating boredom among the players. Changing Tovertafel games during a session was essential to counter this risk, and the staff typically spent no longer than 3 to 5 minutes on each game. Tovertafel games have various difficulty levels, and some were seen as too simple to be played for more than a few minutes. The staff tried to introduce variety by including different types of games within a session, either by manually choosing games or using the Tovertafel’s built-in shuffle function, which moves through games in a randomized order:

I don’t stay on one game for too long, like maybe five minutes. Or I gauge by watching the people around the table and if they’re disengaging or not finding it interesting enough, I’m like, okay, let's switch another game now. You know, to try and give them a bit of variety. And to keep their focus.

Brenda, diversional therapist

#### Using Prompts, Questions, and Storytelling to Make the Games More Engaging

A final creative strategy was the use of different spoken techniques to keep the residents engaged. Sometimes this involved gently prompting the residents. For example, a resident in session 4 was observed to be falling asleep during the game. The staff offered words of encouragement—“Come on George,” “Wake up George”—to keep him interested. In session 5, a resident felt that she “wasn’t doing it right” and stopped interacting with the game. A staff member provided gentle reassurance, saying “No, you’re doing a good job,” which encouraged her to become involved again.

Beyond this gentle prompting and encouragement, the staff tried to keep the residents’ attention on the activity by creatively responding to elements of the games with questions that encouraged interaction. For example, during the Rhymes game, the staff chose to read each rhyme aloud and then asked the residents questions about them. These questions were not part of the game but were added by the staff to make the activity more engaging. In 1 instance, after seeing a rhyme from the song “Rock-a-bye baby,” the staff asked the residents if they knew the rhyme and encouraged them to sing along. During the interviews, the staff explained that this was another technique to maintain the residents’ focus:

To make it work, I keep communicating with the residents and try not to get them off track. Like, you know, just make them stay involved. Otherwise, they’re going to drift off and get sidetracked with the game.

Corina, lifestyle activities coordinator

Other games involved solving puzzles to reveal pictures of places or animals. The staff used these as opportunities to invite conversation about the past, asking the residents questions such as, “Have you ever been to New Zealand?” or “Did you used to have chickens in your garden?” These questions prompted the residents to talk about their lives. Staff members noted that specific games provided inspiration for these conversations, leading to positive outcomes:

What I try to do is to get them to share their stories, so for example when we play the music box game, I might ask them if they had a music box when they were younger. The game with silver polishing is something they would have done when they were younger. You can ask them about that, ask who they had [over] for dinner and what they used to eat.

Brenda, diversional therapist

These behaviors were creative techniques that had been instigated by staff members. They were not a standard part of the Tovertafel games, nor were they part of any training that the staff had received. Rather, they were strategies that staff had found to work well and represented the attempts of staff members to build on the basic elements of the Tovertafel games. This appeared to make the activity more active and engaging, as opposed to one in which the residents passively interacted with the system without understanding what they were doing.

### Theme 3: Staff Creativity Fostered Benefits and Outcomes

This theme highlights 3 benefits arising from the staff-supported enablement of the Tovertafel. These included moments of joy and fun from participating in a physical activity, opportunities for socialization with other residents, and reminiscence prompted by elements of the games.

#### Participating in a Fun Physical Activity

The staff universally viewed the Tovertafel as a positive activity for the residents, and in their view, the benefits outweighed the work involved in setting up and running the technology.

The staff described how keeping the residents engaged was important for enjoyment, otherwise attendees would receive limited value from the activity. Through supporting the residents to use the Tovertafel, we observed that the staff were able to elicit moments of joy and laughter, particularly when playing games that involved considerable physical activity. For example, in 1 diary entry, Brenda noted a positive outcome from the session:

Happy, smiling during the Tovertafel, lots of laughter. There was a change, they were feeling unsure but once participating their mood became more alert and wanted to give it a go.

Brenda, diversional therapist, diary excerpt, session 3

A related benefit was that the Tovertafel encouraged physical activity through the use of the hands and arms. The staff viewed this as a rare opportunity for the residents to engage in gentle exercise, as illustrated by the following comment:

There is a whack-a-mole game, and I like that because we’ve got people with mobility issues and so it’s good exercise for them.

Fiona, enrolled nurse

#### Encouraging Social Interaction Between Residents

The Tovertafel provided an opportunity for the residents to socialize with others in the care home. We observed that, outside of mealtimes or scheduled activities, many of the residents would either be watching television or spending time alone in their room. The residents commented on how they enjoyed the social aspects of the activity:

I think it’s more of a social occasion, when you play games or whatever you play, you can get to know people that way and everybody has different characters, you know what I mean?

George, care home resident

The staff felt that the Tovertafel enabled socialization in a unique way by encouraging residents to interact with people they might not usually engage with:

It gets them together, you know, like having a chat. So, there’s just that bit more interaction. I think it’s way better than the TV. And compared to our trivia games, not everyone gets involved in the trivia. Whereas with [Tovertafel] you can involve most of them. And even if they don't want to play it, at least they might just come and watch. They’re still interacting in some way, you know?

Diane, special care unit nurse

As Diane noted, some residents did not always want to play with the Tovertafel, preferring to sit and watch. Others attended the sessions but were unable to take part because of physical impairment. The staff believed that the Tovertafel still offered a positive opportunity for these residents to socialize, either by watching the activity or by taking on a supporting role in the games. One staff member explained as follows:

The ones who honestly can’t play, I think it’s a good idea, even if they are there to join in and give them something to do, just remember a score or help out because then they are participating and joining in as well. So that's a good thing. You’re not inviting them to sit there and fall asleep. The whole idea of it is to keep them inspired, just to let them know that we're here to have a good time and they’re in a social environment.

Corina, lifestyle activities coordinator

#### Fostering Shared Reminiscence

Finally, the Tovertafel games provided opportunities for shared reminiscence. However, this reminiscence only occurred when the staff prompted the residents to share their stories in response to the games. By asking questions based on the game elements, the staff actively tried to prompt memories about the past. This included topics such as whether the residents used to keep animals in their garden, what they used to make for dinner, and what they typically did to celebrate birthdays—all of which were embodied within different games. This was seen as a particularly positive outcome of using the Tovertafel, as explained by 1 staff member:

Going back in time is good for them to feel good within themselves because it brings back memories. Like this morning, there were some songs that the residents remembered from their younger years. To me that’s a good thing, reminiscing.

Corina, lifestyle activities coordinator

Prompting reminiscence enabled the residents to share their stories and learn about one another. In session 2, we observed that this reminiscence led to a protracted conversation in which a resident shared a story about a country property she helped renovate. The resident later retrieved a photo album from her room and began showing pictures of the renovation works to other residents. Interactions like these enabled the staff to engage in “biography work” [33], which involves learning about residents so that staff can address their needs using more culturally responsive practices. In this way, reminiscence appeared to be beneficial for the residents and the staff members attending each session.

## Discussion

### Principal Findings

#### Overview

This study aimed to understand how the care staff enable the Tovertafel in residential aged care and how this enablement contributes to benefits for residents. Our findings suggest that staff facilitation was crucial for the effective use of the Tovertafel. This began with overcoming barriers to facilitating sessions, including the lack of a dedicated space for play and the need to encourage the residents to become involved. The staff then played a central role in supporting the residents through the activity, using creative techniques to guide the residents through the games and sustain engagement, while paying close attention to the safety of the residents. These actions helped residents gain meaningful benefits from the Tovertafel, which included fun physical activity, socializing with others, and opportunities for shared reminiscence.

#### Staff Enablement of the Tovertafel

Our findings highlight the central role the staff played in enabling the Tovertafel and the importance of creative care strategies in sustaining the participation of the residents. Previous work has argued that creativity can be required when providing enriching technology-mediated activities in care homes [34]. Our findings support this claim by showing how the care staff engaged in creative work during Tovertafel sessions. The Tovertafel provides a range of games, many of which are very simple. Although it may be hoped that the residents can play these games without staff oversight, our findings show that facilitation by the staff was needed to ensure that the residents understood the purpose of the activity, how to play the games, and how to interact with the system. In addition, the staff undertook a variety of creative actions to enhance the engagement of the residents *during* the sessions. The staff were conscious that the residents might choose to disengage from the Tovertafel, given the simplicity and repetitive nature of the games and because some of the residents had limited attention span. Creative actions around the selection and duration of the games helped to mitigate these risks and kept the residents’ focus on the activity. These creative actions, in turn, were likely essential to fostering the benefits and outcomes observed in this study.

However, the involvement of the care staff was also needed to instigate the sessions. This work is arguably less desirable than tasks involving creative care. We observed how staff time was needed to set up the physical space for play—partly a result of where the Tovertafel was placed in the care home. The staff and the residents felt that the activity might be more popular if it were available to use in a dedicated space, such as an activities room. However, previous work has shown that when the Tovertafel was set up in such a space, it was still rarely used by the residents without prompting from caregivers [21]. This implies that facilitation may still be required *during* the use of the Tovertafel, irrespective of where it is located. We also found that staff time was needed to encourage the residents to attend the sessions, given residents’ lack of experience with technology and uncertainty about what to do. These efforts can be viewed as a burden, but they also provided an opportunity for the staff to engage directly with the residents and understand their reasons for nonparticipation. The staff were then able to factor this into the running of the sessions, such as by starting with a simple game to help residents to become comfortable with the technology.

Finally, the care staff were involved in making sure that the residents remained safe during the activity. In residential aged care, many residents are frail or are living with cognitive impairment. This was true for many of the people who participated in the sessions we observed. The care staff ensured that these residents remained safe while using the Tovertafel. The staff believed that the Tovertafel involved some risks for the residents, particularly those who experience dizziness, and so they were vigilant about resident safety. Previous studies have highlighted the need for staff to ensure the safety of residents when using “risky” technologies such as exergames [35] or immersive VR [15,27]. Our study shows that the involvement of the staff was similarly essential to ensure that the residents remained safe when interacting with the Tovertafel.

#### Benefits of Using the Tovertafel

Our findings shed light on benefits arising from the staff-supported enablement of the Tovertafel. First, Tovertafel encouraged physical activity and provoked moments of fun and joy for the residents. It can be difficult to provide stimulating physical activities in residential aged care because of high rates of impairment among the residents [36]. Our observations suggest that the Tovertafel promotes gentle upper-body exercise but can require creative problem-solving and support from the staff to make the activities accessible. We observed this when the staff triggered interactions on behalf of the residents and through their efforts to make the game easier to play by using pieces of foam to extend the reach of the residents.

A second benefit was that the Tovertafel provided an opportunity for the residents to socialize with each other and with the care staff. Although care homes such as the one we studied frequently provide a range of lifestyle activities, opportunities for social interaction can remain scarce [37], contributing to experiences of loneliness [38]. To address these issues, previous work has explored the benefits of technologies that encourage “ludic engagement” through playful interactions between the residents [39,40]. The staff in our study felt that the Tovertafel was similarly beneficial because it brought residents together for playful, face-to-face interaction. This may be hard to achieve with technologies that are single-user focused, such as VR [15]. In addition, the staff noted that the Tovertafel demands cognitive engagement and encourages conversation. This was perceived to be more beneficial than passive activities, such as watching television. It was also perceived as beneficial for the residents who participated as “active audience members” [41], observing while others engaged with the Tovertafel.

A third benefit was that the Tovertafel enabled the staff to prompt reminiscence among the residents. When used effectively, reminiscence can be a positive activity for older people living in residential care [42] and can be supported using technology such as VR [43,44] and digital mapping systems [45]. However, these efforts are also typically single-user focused, and it has been found that while care home residents often engage in reminiscence with family and friends, they do so less frequently with other residents and the care staff [42]. This highlights a role for the Tovertafel in enhancing care: by drawing on elements of the games in a social setting, the staff were able to prompt reminiscence by encouraging residents to share their life stories. Previous research has shown positive outcomes when care staff are involved in reminiscence because they can develop closer relationships with the residents and enhance their care work [46]. We saw this in our study when the staff used the Tovertafel to engage in “biography work,” enabling them to learn new things about the residents [33]. Overall, these findings highlight how the Tovertafel can gently prompt reminiscence in ways that benefit both the residents and care staff. They also point to a positive feature of the Tovertafel games, whereby the open-ended design of most games allows for creative facilitation by the staff [34].

However, it is important to note that these benefits were contingent on the significant involvement of the care staff to enable the participation of the residents. On the basis of our findings, it is unlikely that similar outcomes will be achieved if the residents are not supported to use the Tovertafel. This may demand additional staff time in cases where the staff are not already rostered to support enrichment activities. The need for enablement by the staff, coupled with the high cost of the technology itself, should be a key consideration when deciding whether to invest in the Tovertafel. This study suggests that the Tovertafel can be an enriching small-group activity that can meet the needs of individual residents while affording opportunities for creative care work. However, as the staff had discovered at the home we studied, the Tovertafel should be adopted as part of a diverse activities program that includes large- and small-group activities; those that use digital technology; and those that encourage investment in physical infrastructure, such as gardening and interest-based hobbies [10,13].

### Organizational Considerations for the Use of the Tovertafel

Our findings highlight several considerations that may inform decisions to implement the Tovertafel within residential aged care.

First, the Tovertafel is a high-cost investment that requires a significant amount of ongoing support to maintain use of the resource. The level of involvement from the staff required to effectively facilitate the Tovertafel is a potential barrier, especially in residential care homes where staff shortage is high, and priority is often given to meeting the personal care needs of the residents [2]. Aligning with previous work [21], our findings emphasize the competencies required by the staff to encourage the residents to use the Tovertafel, especially when those residents have cognitive impairment. This requires a team effort as the Tovertafel appears to work best with support from at least 1 staff member who can facilitate the engagement of the residents and maintain their attention.

Although training staff about the benefits and features of the Tovertafel is important [47], our findings demonstrate that training the staff *how* to creatively facilitate sessions may be even more valuable for maximizing its impact. This type of training is unlikely to be attained via reading manuals or resources and is better acquired through hands-on experience—as was the case with our participants. Training staff on good practices related to the Tovertafel could help with “onboarding” new staff to deliver the activity, given that staff turnover is a known barrier to the successful implementation of technology [16]. Alternatively, training could focus on developing the key competencies of the staff required for facilitation (particularly communication skills), allowing them to facilitate a range of activities [48].

In this study, we observed that facilitation skills were modeled to new staff by experienced staff and mentors. For instance, training typically involved a staff member demonstrating effective strategies while explaining the reasons for each approach aloud. However, this approach is likely to be an obstacle in residential aged care because of high staff turnover and limited staff-family training. Therefore, we recommend that staff receive general communication partner training, which can equip people with the skills required to respond effectively to the needs of the residents and to facilitate communication strategies [49]. Such training focuses on building interpersonal skills and fostering effective interactions, enabling staff to better engage with residents and enhance their facilitation capabilities.

Our study highlighted the need for an appropriate space to enable use of the Tovertafel. In the home we studied, the Tovertafel was situated in 2 specialized care areas: one for people with physical care needs and another for residents with severe cognitive impairment. However, in both locations, the Tovertafel was used in the main dining spaces, where the residents could not always see the projected images clearly. The need to set up the Tovertafel and pack it away meant it was not readily available for spontaneous activity or facilitation by family members and visitors. The residents and the staff felt that the system might be used more often if it were set up in a dedicated space, such as an activities room. This would enable the residents to access the resource with the staff as well as other available facilitators [21].

Third, appropriate scheduling of the Tovertafel needs to be considered to prevent disinterest among residents. According to the staff we interviewed, the Tovertafel had been dormant for some time before this study. This was because the residents had become uninterested in using it, leading to low attendance at the sessions. At the time of this study, staff were reintroducing the Tovertafel as a new activity to elicit interest. However, we observed that the Tovertafel was not well known among the residents compared to other activities. The Tovertafel was challenging to describe and required the staff to promote the benefits of the activity, relying on the residents’ prior knowledge and interest in technology. Thus, the staff found it difficult to encourage residents to participate. This is challenging because the Tovertafel is an expensive technology and may be best deployed with larger numbers of residents so that it provides a return on investment.

Studies have reported that facilitators for promoting participation in technology-based activities can include communicating the benefits of use, building knowledge and confidence in using technology, and offering support during its use. In line with these findings, we offer some practical strategies for promoting interest in the Tovertafel: (1) communicating benefits; organizing informative sessions, where staff demonstrate the technology and its benefits, may increase residents’ familiarity and engagement; (2) building knowledge and confidence in using technology; sharing success stories and positive outcomes from previous sessions can serve as motivation for residents to participate; and (3) offering support; a buddy system could be established, pairing residents who are comfortable with technology with those who may be hesitant, fostering a supportive environment for participation. These strategies should be co-designed with residents and staff to ensure they are suitable for this context.

### Limitations and Future Work

One limitation of this study is that we were only able to capture data about a handful of Tovertafel sessions, which occurred during a period of 4 weeks with a small number of residents. This may have constrained our observations on the breadth of strategies used by the staff. This limitation was mitigated through the use of diaries that were completed by the staff in additional sessions. Future studies can explore the impact of varying session lengths on the engagement of residents and strategies used by the staff to gain additional insights into effective facilitation techniques. Future research could also monitor the use of Tovertafel over the longer term to ascertain whether sustained engagement by both the staff and the residents occurs. Given the high cost of the device, ongoing use would be required for it to be cost-effective; otherwise, financial resources might be better spent on other beneficial activities and programs with higher engagement over the longer term.

Second, we focused on a single care home in Australia. However, gaining access to residential aged care for research is challenging, and studies are often based on a single site [8,10]. In addition, the Tovertafel is still not in widespread use, making it challenging to identify appropriate study sites to compare findings. Our findings have value in expanding the knowledge base about the Tovertafel and contribute to an improved understanding of the role that staff play in facilitating technologies that are hard for residents to use independently. For example, the benefits of enjoyment and physical activity from the Tovertafel align with a previous study conducted in the United Kingdom [21]. Nevertheless, further research should be conducted to identify context-dependent benefits and evaluate the transferability of our findings.

This study offers limited insight into the views of the residents. Many of the residents observed in our study did not have the cognitive capacity to consent to interviews. The residents who were interviewed had little to say about the Tovertafel, possibly because they were not fully aware of its potential features or because of their brief interaction with it. Future work should aim to provide a deeper understanding of the experiences of the residents. This information could then be used to inform best practices for care staff.

### Conclusions

This study investigated the role of the staff in facilitating the effective use of the Tovertafel in 1 Australian residential aged care home. We found that creative strategies were needed to encourage the residents to use the Tovertafel and to support the participation of the residents in the activity. Beneficial outcomes included experiencing fun and joy, social interaction, and reminiscence. The staff proactively mitigated challenges in engagement, which included barriers to access and the adverse reactions that the residents may have to the experience. Aligning with previous research on the use of technology for enrichment in aged care [11,15,17], this study highlights the pivotal role of care staff in the adoption and use of digital technology, meaning that aged care providers need to account for staff involvement when deciding whether to adopt the Tovertafel.

## Appendix

Multimedia Appendix 1 Design of the feedback diary completed by the staff participants.

## Abbreviations

PICF: participant information sheet and consent form
VR: virtual reality
