# Supplementary material for: Staff Enablement of the Tovertafel for Enrichment in Residential Aged Care: Field Study
Source: JMIR Aging. 2025 Aug 12;8:e67919. doi: 10.2196/67919 (PMC12381539; doi:10.2196/67919)
Supplement: Multimedia Appendix 1 [file aging_v8i1e67919_app1.docx]

## Appendix A – Diary Design

| Name |  | | |
| --- | --- | --- | --- |
| Day |  | | |
| Time |  | AM / PM ? |  |
|  | | | |
| Who was involved in using the Tovertafel today? (Which residents and staff?) | | | |
|  | | | |
| What observations did you make about residents’ mood before and after using the Tovertafel? Was there any change? | | | |
|  | | | |
| What worked well during the last Tovertafel session? | | | |
|  | | | |
| What challenges did you face when facilitating the last Tovertafel session? Was there anything that didn’t work well? | | | |
|  | | | |
| Is there anything else you would like to add about your experience of using the Tovertafel? | | | |
|  | | | |
